# Reducing shade avoidance responses in a cereal crop

**DOI:** 10.1093/aobpla/plx039

**Published:** 2017-08-08

**Authors:** Wibke Wille, Christian B Pipper, Eva Rosenqvist, Sven B Andersen, Jacob Weiner

**Affiliations:** 1 Department of Plant and Environmental Sciences, University of Copenhagen, DK-1871 Frederiksberg, Denmark; 2 Department of Public Health, University of Copenhagen, DK-1014 Copenhagen, Denmark

**Keywords:** Cereals, forward screening, induced mutations, mutant screening, plasticity, red:far-red

## Abstract

Several researchers have hypothesized that shade avoidance behaviour is favoured by natural selection because it increases the fitness of individuals. Shade avoidance can be disadvantageous for crops, however, because it reduces allocation of resources to reproductive yield, increases the risk of lodging and reduces weed suppression. One approach to develop varieties with reduced shade avoidance and enhanced agronomic performance is by inducing mutations followed by phenotypic screening. We treated spring wheat seeds with ethyl methanesulfonate and screened the seedlings repeatedly under green filters for plants showing reduced elongation of the first leaf sheath and second leaf lamina. The shade avoidance responses of five promising mutant lines were further compared to non-mutated plants in a climate chamber experiment with added far-red light. Two of the selected lines displayed significantly reduced elongation under all light treatments while two lines showed reduced elongation only in added far-red light. The most promising mutant line did not differ in height from the non-mutated cultivar in neutral light, but elongated 20.6% less in strong far-red light. This traditional forward approach of screening mutagenized spring wheat produced plants with reduced shade avoidance responses. These mutants may generate new molecular handles to modify the reaction of plants to changes in light spectral distribution in traditional and novel cultivation systems.

## Teaser

Sun-loving plants react to changes in light quality caused by neighbouring plants via “Shade Avoidance” responses, including vertical elongation, upward orientation of leaves and reduced branching/tillering. Such responses are favoured by natural selection because they increase the fitness of individuals, but can be disadvantageous for crops. We took a step towards the development of varieties of wheat with reduced shade avoidance by inducing mutations followed by phenotypic screening. The most promising mutant line did not differ in height from non-mutated cultivars under normal conditions or neutral shade, but elongated much less in strong far-red light.

## Introduction

Plants possess sensory mechanisms to detect changes in ambient light caused by adjacent vegetation. Leaf chlorophylls and carotenoids absorb mostly red (R) and blue light. Thus, light that is transmitted through vegetation is depleted in red and strongly enriched in far-red (FR; Smith 2000; Franklin and Whitelam 2005). Even before direct shading takes place, FR light reflected from neighbouring plants lowers the R:FR light ratio, acting as an early signal of neighbour proximity (Casal *et al.* 1986; Ballaré *et al.* 1990). Later, the depletion of red and blue light signals a transition from neighbour detection to real competition (de Wit *et al.* 2016). The natural R:FR ratio varies from 1 to 1.2 in sunlight above a canopy, gradually decreasing to <0.2 under a dense canopy (Gundel *et al.* 2014). Changes in the R:FR light ratio are detected by signal-transducing photoreceptors, phytochromes, and result in morphological responses termed the ‘shade avoidance syndrome’. These responses include a strong elongation of stem-like organs, upward orientation of leaves (hyponasty), reduced branching or tillering and early flowering (Smith and Whitelam 1997; Ballaré and Casal 2000; Franklin 2008).

While shade avoidance increases the fitness of an individual in a crowded plant population (Schmitt *et al.* 1995), helping it to reach the canopy and avoid being shaded by neighbours, it is likely to reduce population yield of crops, as the elongation of the plants is achieved at the expense of leaf area, tillering and root growth (Casal *et al.* 1986; Kasperbauer and Karlen 1994; Ruberti *et al.* 2012; but see Ballaré *et al.* 1991). Reduced allocation of resources to roots has a negative impact on the anchoring capacity of the crop plants and increases the risk of lodging (Sparkes and King 2008) and water depletion. In line with this, several studies have suggested that reduced shade avoidance responses could increase cereal crop yields (Smith 1992; Holt 1995; Sawers *et al.* 2005; Carriedo *et al.* 2016).

In addition to these direct effects, we have argued that the shade avoidance response is detrimental to a crop’s ability to suppress weeds at high density. Cereals have greater potential to suppress weeds than previously thought and this potential can be realized by increasing the density and spatial uniformity of the crop (Weiner *et al.* 2001). While high plant density offers increased weed suppression, the earlier competition triggers earlier shade avoidance responses. The upward orientation of the plant leaves and reduced tillering under shade avoidance allow light to penetrate deeper into the canopy, thereby reducing the shading of weeds by the crop population. Consequently, we hypothesize that reduced shade avoidance by the crop will increase weed suppression at high density (Weiner *et al.* 2010), because shade avoidance is an individual, defensive strategy to avoid being shaded, while weed suppression at high density is an offensive, group strategy to shade weeds when they are still small.

Thus, reduced shade avoidance offers a promising target for breeding, since wild type shade avoidance is an evolutionary mechanism favoured by natural selection because it benefits the individual, but at a cost for the population (Weiner *et al.* 2010; Denison 2012). The aim of this study was to explore the possibility of developing spring wheat plants with reduced shade avoidance responses. As knowledge of the genetic control of shade avoidance in wheat remains limited, we used a traditional forward genetic screening of EMS mutagenized material. Such an approach could generate new phenotypes with altered light signalling downstream of the primary light receptors and provide new molecular handles to alter plant reactions to changes in the light spectrum.

## Methods

### Plant material and growth conditions

The glasshouse experiments were conducted in 2009–12 (Table 1) at Frederiksberg (55°41′N, 12°32′E) and the climate chamber work was located at Taastrup, University of Copenhagen, Denmark. The initial seed material consisted of selfed and bulk harvested M2 seeds from six spring wheat cultivars (Amaretto, Vinjett, Triso, CPBT W93, Dragon and Dacke), treated with three concentrations of ethyl methanesulfonate (0.038 M, 0.075 M and 0.15 M; M0880-100G, Sigma, Copenhagen, Denmark). Seeds were sown in trays (TEKU JP3050/230H and VEFI PK60) or in pots (Pöppelmann Plastik Skandinavien Aps, Odense, Denmark) in sphagnum substrate (Pindstrup Substrate 1&7; Pindstrup Mosebrug A/S, Ryomgaard, Denmark) and watered with 2 g of Topsin WG (Tiofanatmetyl 700 g kg^−1^, Nordisk Alkali AB, Malmoe, Sweden) dissolved in 10 L of water, to deter fungal infections. Seedlings were sub-irrigated (greenhouse) or drip-irrigated (growth chambers) with a full nutrient solution (‘Gødningsblanding’, Plant Facilties, Department of Plant and Environmental Sciences, University Copenhagen) at pH 5.5. The glasshouses provided supplementary light from 600 W SON-T Green Power lamps (E40, Philips, Eindhoven, Netherlands) 16 h a day, unless otherwise stated. The day/night temperatures were 15/12 °C and ventilation started when the temperature increased 3 °C above the set point.

**Table 1.** Summary of the selection steps to obtain mutants with reduced shade avoidance.

Experiments, *R:FR*	Time	Seeds	Mutants	Methods	Selected
Greenhouse screening 1, *R:FR 0.65*	2009	M2	1000000	Bulk trays, phenotype screening, green filters, reduced red and blue light	13850
Greenhouse screening 2, *R:FR 0.65*	2009	M3	7200	Mutant lines, phenotype screening green filters, reduced red and blue light	248
Greenhouse selection 1, *R:FR 1.1 and 0.16*	2010	M4	163 lines	Measured traits, green and neutral filters, reduced red and blue light	36
Greenhouse selection 2, *R:FR 1.1 and 0.16*	2011	M5	36 lines	Measured traits, green and neutral filters, reduced red and blue light	5
Climate chamber, *R:FR 2.5, 0.9 and 0.4*	2012	M5	5 lines	Measured traits, far-red LED modules, added far-red light	2

Many filters have been used in earlier shade avoidance experiments (Hiraut-Bron *et al.* 2001; Causin and Wulff 2003; Pierik *et al.* 2005; Weijschedé *et al.* 2006). In this study, LEE Pale green 138 filters were used for the initial visual screening in early spring, and LEE Fern green 122 filters and Lutrasil Pro 23 (neutral shade) were used in the subsequent experiments, carried out in the summer (LEE filters, Andover, UK and Lutrasil Pro 23 g m^−2^, Freudenberg Vliesstoffe KG, Germany). The Pale green 138 filter had a triple transmission peak in the range 495–555 nm, the Fern green 122 filter a double transmission peat in the range 510–535 nm, while the neutral shade reduced all wavelengths approximately equally in the range 400–750 nm.

### Screening 1—visual selection of divergent phenotypes under green shading

Approximately 1 million mutated M2 seeds were sown in trays under green filters (LEE 138) that reduced the R:FR ratio (655–665:725–735 nm) from 1.1 to 0.65, and photosynthetic active radiation (PAR) by 40 %. Four weeks after sowing, at a 3–4 leaf stage, plants were screened for individuals that did not display characteristic traits for shade avoidance, that is rapid elongation of leaf sheaths and laminae, reduced chlorophyll concentration, leaf hyponasty and reduced tillering. The selected plants had shorter leaf sheaths and/or laminae and darker green leaves than other plants, and some had started tillering. These selected mutants were transplanted into 10 cm pots and moved outdoors in April. At maturity, the self-pollinated plants were harvested and threshed, and grains produced by each plant were kept separately.

### Screening 2—visual selection for lines with homozygous mutations under green shading

In October 2009, the M3 seeds obtained from the previous screening were sown in rows of 10 seeds per parent plant on trays placed under green filters (LEE 138). The plants were screened as before, 4 weeks after sowing, at the 3–4 leaf stage, selecting for mutations for reduced elongation responses under green shading. The selected lines were transplanted into 10 cm pots, left to self-pollinate and later harvested and threshed.

### Selection 1—selection for extreme elongation behaviour in low R:FR light

In June 2010, the shade avoidance responses of 163 selected mutant lines were compared to the four non-mutated cultivars they originated from. Two sets of 6 seeds per mutant line and 30 seeds per non-mutated cultivar were sown in 10 cm pots that were placed on two 16 m^2^ tables in the glasshouse. One table was covered with a LEE Fern green 122 filter that reduced the R:FR ratio from 1.13 to 0.16 and PAR by 60 %. The other table was covered with several layers of neutral Lutrasil Pro 23 fabric to reduce PAR by 60 % without changing the R:FR ratio. No supplemental light was used during the experiment. Both tables were divided into six blocks. Within each block, one plant of every mutant line and two plants of the non-mutated cultivars were placed in a random order on trays, 10 cm apart. Border plants were positioned around the table edges to reduce edge effects.

In two pilot experiments conducted in 2009 and 2010, the length of the first leaf sheath (soil surface to first leaf ligule) and the second leaf lamina (stalk to the tip of the leaf) resulted in the most reliable measures of early elongation responses. In this screening experiment, these measurements were taken once a week for 3 weeks, starting 3 weeks after sowing. It is known that reduced R:FR ratios suppress tillering in grasses (Casal *et al.* 1986; Evers *et al.* 2006). As tillering may enhance weed suppression at early stages, the number of tillers was recorded at the last measurement. Three weeks after the first sowing the experiment was repeated in all details.

The mutant lines were ranked according to their elongation response (difference in the length of the first leaf sheath and of the second leaf lamina between neutral and green shading), using random effects estimates (Robinson 2001). Based on the ranking of the lines and a phenotypic evaluation of the mature mutant plants, vigorous and seed-setting lines with a high variance in elongation responses were selected for the next experiment. To reduce the number of dwarf mutations and the number of lines, so they could be handled in pot experiments, lines were considered only if they had a final mean plant height of at least 60 % of the mean of the non-mutated cultivars.

### Selection 2—selection for mutant lines with reduced elongation in low R:FR light

In June 2011, the selected 36 mutant lines were grown once more under green (LEE122) and neutral shade (Lutrasil Pro 23), and their elongation responses compared to the non-mutated cultivars. According to the availability of seeds, two sets of 5–30 seeds per each mutant line and 30 seeds per non-mutated cultivar were sown in 10 cm pots and placed in a fully randomized order on two glasshouse tables. No supplemental light was used in the experiment. Measurements were conducted exactly as the year before and the experiment was repeated in all respects 3 weeks after the sowing of the first experiment. The mutant lines were again ranked according to the difference in the length of the first leaf sheath and second leaf lamina under neutral versus green shading, using random effects estimates (Robinson 2001). Based on the ranking of the lines and a phenotypic evaluation of the mature mutant plants, four vigorous, seed-setting lines with very low elongation responses and one interesting strong tillering phenotype were selected for further testing.

### Selection for mutant lines with reduced elongation in added FR light

In November 2011, the shade avoidance responses of the five most promising mutants were measured in a reduced R:FR light environment. Instead of reducing the R light with green filters, the plants were exposed to two levels of added FR light. The experiment was conducted in three climate chambers with a 3.3 m^2^ growing area (Conviron Walk-In, PGV 56, Winnipeg, Canada) equipped with eight metal-halide lamps (Osram Powerstar 400W/D Pro HQI-BT, Osram, Munich, Germany). In the first chamber, a R:FR light ratio of 0.4 was supplied by installing 12 FR LED production modules (Philips GreenPower LED150, 39 µmol m^−2^ s^−1^ for 33W, Philips, Eindhoven, the Netherlands) under HQI-BT lamps (as used in Pierik *et al.* 2005), in the second chamber a R:FR ratio of 0.9 was supplied using four modules. In a third chamber, the R:FR light ratio was 2.5 without added FR light. Wooden dummies were used to compensate for the different numbers of LED modules in the three chambers and to give even shade and PAR levels that were as close as possible viz: 348 ± 20.3, 327 ± 15.7 and 344 ± 20.1 µmol m^−2^ s^−1^ (average of 20 positions ± SD) in the three climate chambers, respectively. Measurements were taken with an AvaSpec-2048 spectrometer (Avantes, Apeldoorn, the Netherlands).

Ten 13 cm pots with three seeds per pot for each of the five mutant lines and their two parent cultivars were prepared for each chamber. After germination, the plants were thinned to one seedling per pot. The pots of each line were evenly distributed in the chambers on four trolleys and the order of the trolleys was changed three times a week. The plants received drip irrigation with full nutrient solution and were subjected to 16/8 h of light/dark period and 20/16 °C day/night temperatures.

Measurements of all leaf sheath and lamina lengths were taken once a week for 5 weeks after germination, and tillers were counted after the last measurement. The level of spike development was recorded three times a week for 10 weeks and disk samples for chlorophyll measurements were taken 11 weeks after sowing. After 14 weeks, the plants were harvested. From each chamber five plants per line were randomly taken for leaf area determination by scanning the leaves of five tillers per plant (LI-COR 3100 Area meter, LI-COR, Lincoln, NE). All plant material was dried in 80 °C for 24 h, after which leaf, stem and spike biomass were weighed.

### Chlorophyll extraction

Four disks of 5 mm or two disks of 7 mm in diameter (depending on the width of the leaf) were punched from the centre of the third leaf of each plant. The samples were weighed, and placed in vials containing 3 mL dimethylformamide (DMF, Sigma–Aldrich, Brøndby, Denmark). The vials were placed in a dark fridge, in 4 °C for 24 h to extract chlorophyll. The absorbance of the chlorophyll solution was then measured with a spectrophotometer (Unicam UV1, Unicam, Cambridge, UK) at 647.0 and 664.5 nm, and chlorophyll *a* and *b* concentrations were calculated according to Inskeep and Bloom (1985).

### Statistical analysis

Ranking of mutant lines with reference to treatment response was done by means of mixed linear models. Specifically, elongation of leaf sheaths and leaf laminae were modelled including main effects of R:FR ratios, parent/mutant status, cultivar and time of recording as well as random effects of line and line within treatment. From these models, random effects estimates of line within treatment were obtained by restricted maximum likelihood (REML) estimation. The change in random effects estimates within line due to treatment was used to rank the lines (Robinson 2001).

For the chosen mutant lines and their parental lines, leaf sheath and leaf lamina sizes above zero at different stages and over time were analysed with mixed linear models. This model included a combined effect of treatment and line adjusted for the combined effect of stage and time. A random effect of plant was included in the model to accommodate repeated measurements of the same plants. *Post hoc* comparisons of parents and mutants were based on REML estimation and adjusted for multiple testing by means of the single-step procedure (Hothorn *et al.* 2008). Reported *P*-values correspond to *Z*-tests and were evaluated at a 5 % significance level. All analyses were performed in R version 2.15 (www.r-project.org).

## Results

### Screening 1 and 2

The initial screening of approximately 1 million mutated seedlings under green filters resulted in the selection of 13850 individuals with reduced apical elongation, darker and broader leaves, shorter than average leaf sheaths or early tillering. Of the selected plants, 7200 individuals produced seeds which were sown in rows under a green filter to obtain mutant offspring with short stature phenotypes deviating from the parent cultivars. From these rows 248 lines were chosen and evaluated at maturity for vigour and seed set, resulting in 163 mutant lines used in subsequent experiments (Table 1).

### Selection 1 and 2 with reduced red light

In the first selection experiment, 163 mutant lines and 4 non-mutated original cultivars were placed under green and neutral filters to select for mutant lines with different elongation responses to shading. In this experiment, most of the plants responded to green shading with strong elongation: the length of the first leaf sheath was on average 7.5 mm (*P* < 0.001) longer and the second leaf lamina 9.33 mm (*P* < 0.001) longer in plants grown under green than under neutral shade. The plants under neutral shade had more tillers (*P* < 0.001) than under green shade (Table 2). No significant difference was detected in the average elongation or tillering responses between the cultivars and the between the 163 mutants due to strong variation in responses of the mutants to reduced red light. After ranking the mutant lines according to their elongation response (difference between the random effects estimates of the first leaf sheath and second leaf lamina length between green and neutral shading) and evaluating the ranked lines at maturity for vigour and seed set, and discarding individuals shorter than 60% of the final height of the original cultivars, 36 mutant lines with extremely strong and extremely weak shade avoidance responses were selected for the second experiment.

**Table 2.** Selection experiments in 2010 and 2011, in which mutated spring wheat plants and their parental cultivars were placed under green and neutral filters to study the elongation responses of their first leaf sheath and second leaf lamina and their tillering behaviour to vegetative shading.

	Difference	Estimate	SE	*z* value	Pr (>|z|)	Confidence interval	
						lower	upper
Selection 1 (2010)							
Length of 1st leaf sheath (mm)	Neutral–green shade	−7.50	0.42	−17.82	<0.001	−8.33	−6.68
	Cultivar–mutant	14.17	7.28	1.95	0.264	−0.10	28.45
Length of 2nd leaf lamina (mm)	Neutral–green shade	−9.33	1.60	−5.83	<0.001	−12.47	−6.19
	Cultivar–mutant	74.41	31.79	2.34	0.138	12.09	136.73
Number of tillers	Neutral–green shade	0.50	0.03	17.38	<0.001	0.44	0.56
	Cultivar–mutant	0.17	0.07	2.36	0.087	0.03	0.31
Selection 2 (2011)							
Length of 1st leaf sheath (mm)	Neutral–green shade	−4.07	0.51	−7.91	<0.001	−5.08	−3.06
	Cultivar–mutant	3.32	4.19	0.79	0.983	−4.89	11.53
Length of 2nd leaf lamina (mm)	Neutral–green shade	−9.54	2.30	−4.15	<0.001	−14.04	−5.03
	Cultivar–mutant	21.55	21.98	0.98	0.939	−21.54	64.63
Number of tillers	Neutral–green shade	0.52	0.04	13.06	<0.001	0.44	0.60
	Cultivar–mutant	0.00	0.16	−0.03	1.000	−0.31	0.30

In the second selection experiment, the elongation responses of these 36 mutant lines and the 4 non-mutated cultivars were ranked to select for the mutant lines with the lowest shade avoidance responses to reduced red light. In this experiment, most of the plants responded to the low R:FR ratio under the green filter with strong elongation of the first leaf sheath (4.07 mm, *P* < 0.001) and the second leaf lamina (9.54 mm, *P* = 0.001), and with reduced tillering (*P* < 0.001, Table 2). After ranking the mutant lines according to their elongation responses (Table 3) four promising Amaretto mutant lines (A9, A20, A48 and A151) were selected because they displayed less elongation of the second leaf lamina. In two of these mutants, the first leaf sheaths also elongated less under green shading than non-mutated Amaretto whilst having only a slightly reduced mature plant height. An interesting mutant of Triso (T104) was also chosen for further testing due to its strongly increased tillering under green shade, even though it elongated more than the non-mutated Triso (Table 3).

**Table 3.** Mutant lines from the selection experiment in 2011, ranked according to the difference in elongation of the plants’ first leaf sheath (a) and the second leaf lamina length (b) under green and neutral shade using random effects estimates.

(a) First leaf sheath					(b) Second leaf lamina				
Cultivar	Line	Type	Difference green–neutral	Plant height (mm)	Cultivar	Line	Type	Difference green–neutral	Plant height (mm)
Amaretto	151		−1.27	678	Amaretto	151		−1.18	678
	48		−0.46	668		48		−0.95	668
	164	Parent	−0.05	730		133		−0.91	698
	133		−0.02	698		150		−0.47	710
	20		−0.01	732		9		−0.34	670
	9		0.14	670		20		−0.27	732
	128		0.42	641		128		0.00	641
	50		0.44	610		6		0.57	764
	150		0.46	710		50		0.70	610
	39		0.58	440		5		0.85	500
	130		0.68	723		164	Parent	0.89	730
	6		0.84	764		39		1.00	440
	5		1.35	500		130		1.43	723
Vinjett	140		−1.79	599	Vinjett	157		−1.61	589
	157		−0.85	589		153		−1.09	618
	142		−0.38	631		139		−0.57	619
	153		0.04	618		140		−0.25	599
	165	Parent	0.24	713		142		−0.17	631
	159		0.27	688		165	Parent	0.35	713
	65		0.73	578		159		0.50	688
	139		1.42	619		65		1.34	578
	55		1.47	569		59		1.36	323
	59		2.58	323		55		1.81	569
Triso	160		−1.05	500	Triso	162		−1.81	396
	166	Parent	−0.89	688		105		−1.08	619
	162		−0.57	396		103		−0.96	485
	107		−0.49	663		95		−0.62	606
	103		−0.42	485		166	Parent	−0.38	688
	145		−0.41	460		160		−0.14	500
	95		−0.32	606		107		−0.07	663
	93		−0.27	532		93		0.02	532
	105		−0.12	619		97		0.43	557
	97		0.10	557		163		0.79	539
	101		0.82	677		145		1.14	460
	104		1.14	636		101		1.61	677
	163		1.52	539		104		1.73	636
CPBT-W93	115		−2.42	548	CPBT-W93	167	Parent	−1.48	613
	167	Parent	−1.59	613		115		−1.43	548
	117		−1.30	474		117		−0.85	474
	119		−0.57	636		119		0.12	636

### Climate chamber experiment with added far-red light

**Figure 1.**
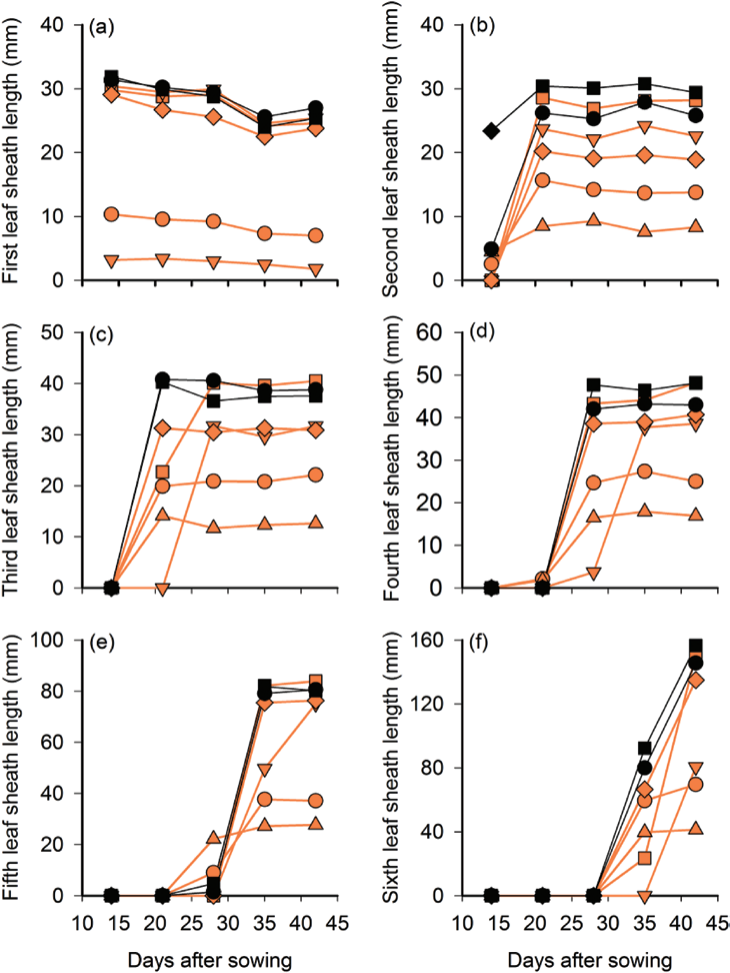
Leaf sheath growth patterns of five spring wheat mutant lines and their non-mutated parent cultivars in added FR light (R:FR 0.4). Mutants depicted in orange and non-mutated cultivars in black lines and symbols. Red circles: A9, red squares: A20, inverted triangles: A48, triangles: T104, diamonds: A151, black circles: Amaretto, black squares: Triso.

When the five mutants showing reduced shade avoidance behaviour and the non-mutated cultivars were placed in climate chambers with supplemental FR light, four of the five mutants showed reduced leaf sheath elongation in the lowest R:FR ratio compared to the non-mutated cultivars (Fig. 1). Differences in leaf sheath length between mutant and wild type was most pronounced for mutant lines A9 and T104 (20.98 and 31.30 mm) but statistically significant also in lines A48 and A151 (10.84 and 6.61 mm, Table 4).

**Table 4.** Leaf sheath and lamina lengths of five selected spring wheat mutant lines compared with non-mutated cultivars of their origin when grown with enhanced FR light, *n* = 10.

	R:FR	Estimate (mm)	SE	Pr (>|z|)	Confidence interval	
					Lower	Upper
Leaf sheaths						
A9—Amaretto	2.5	−16.69	1.570	<0.001	−19.77	−13.62
	0.9	−17.24	1.573	<0.001	−20.32	−14.15
	0.4	−20.98	1.579	<0.001	−24.07	−17.88
A20—Amaretto	2.5	1.67	1.583	0.987	−1.43	4.77
	0.9	1.42	1.554	0.997	−1.63	4.46
	0.4	−4.03	1.521	0.105	7.01	−1.05
A48—Amaretto	2.5	−1.31	1.647	0.999	−4.54	1.92
	0.9	−6.15	1.608	0.002	−9.30	−2.99
	0.4	−10.84	1.581	<0.001	−13.94	−7.74
T104—Triso	2.5	−20.94	1.601	<0.001	−24.08	−17.81
	0.9	−27.94	1.577	<0.001	−31.03	−24.85
	0.4	−31.30	1.561	<0.001	−34.36	−28.24
A151—Amaretto	2.5	−3.77	1.577	0.203	−6.86	−0.68
	0.9	−4.22	1.539	0.082	−7.23	−1.20
	0.4	−6.61	1.514	<0.001	−9.58	−3.64
Leaf laminas						
A9—Amaretto	2.5	−44.23	5.500	<0.001	−55.01	−33.45
	0.9	−33.05	5.549	<0.001	−43.93	−22.18
	0.4	−38.65	5.531	<0.001	−49.49	−27.81
A20—Amaretto	2.5	−17.86	5.531	0.016	−28.70	−7.02
	0.9	−11.07	5.503	0.391	−21.85	−0.28
	0.4	−10.15	5.471	0.507	−20.87	0.58
A48—Amaretto	2.5	−60.13	5.643	<0.001	−71.19	−49.07
	0.9	−48.13	5.618	<0.001	−59.14	−37.12
	0.4	−58.55	5.547	<0.001	−69.43	−47.68
T104—Triso	2.5	−85.27	5.475	<0.001	−96.00	−74.54
	0.9	−92.43	5.431	<0.001	−103.07	−81.79
	0.4	−76.86	5.428	<0.001	−87.50	−66.22
A151—Amaretto	2.5	−13.72	5.539	0.146	−24.58	−2.86
	0.9	−7.24	5.492	0.884	−18.01	3.52
	0.4	−13.17	5.448	0.169	−23.84	−2.49

**Figure 2.**
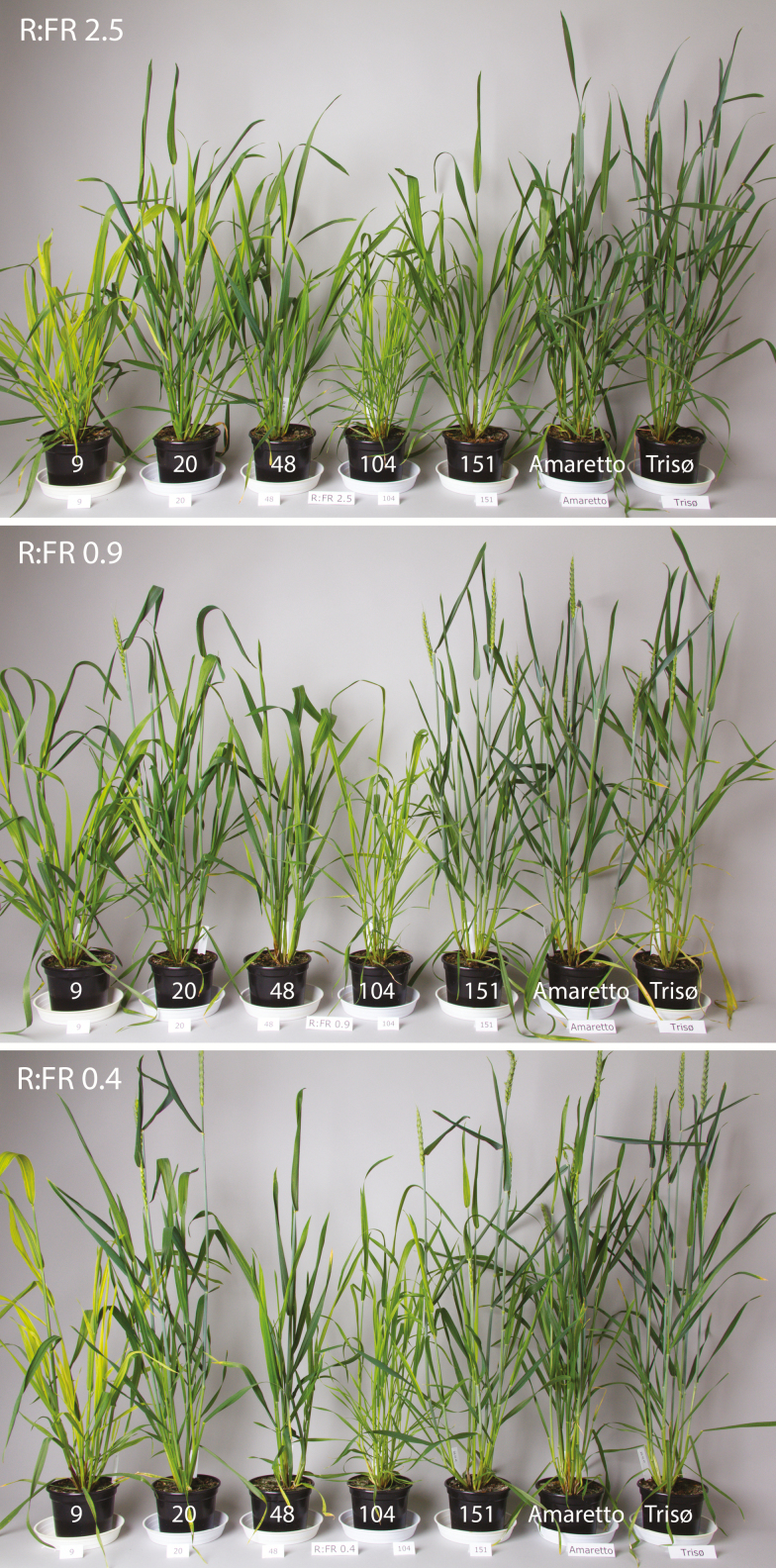
Selected mutant lines A9, A20, A48, T104 and A151 and two non-mutated cultivars Amaretto and Triso, grown in R:FR 2.5, 0.9 and 0.4. Photos were taken 8 weeks after sowing. Plants grown in R:FR 0.4 show a faster development, earlier spike emergence and anthesis than plants in R:FR 2.5.

The mutants A48 and A151 showed no significant difference in leaf sheath growth from the non-mutated lines in R:FR 2.5, but displayed signs of reduced elongation at R:FR 0.9 and responded with significantly reduced elongation to low (0.4) R:FR light (68.9 % and 20.6 % total reduction in elongation compared to parental lines, Table 4, Fig. 2). Although these mutants reached a slightly shorter final height in near neutral light (R:FR 0.9) than did non-mutated cultivars (A48: 11.4 % and A151: 6.8 %, Table 6) their reduced elongation was clearly a gradual response to increased shade (Table 4). The leaf lamina responses of these two mutants differed. While the leaf laminae of A48 were shorter than those of non-mutated plants in all light treatments (*P* < 0.001, Table 4) those of A151 did not differ from the non-mutated plants in any of the treatments. The lack of a gradual response to shading in the length of the second leaf lamina in added FR light, while evident in the green filter experiment (Table 3), may be due to the stronger reduction of the R:FR ratio under the green filters (R:FR 0.16) than in the climate chambers (R:FR 0.4)

In near neutral light (R:FR 0.9), A48 had, on average, a 10.4 % larger leaf area than non-mutated Amaretto, in spite of having a fewer tillers (10.5 vs. 15.1). It also had thick, dark green leaves and the ears emerged on average 11 days later than those of the non-mutated Amaretto (Table 5). The mutant A151 on the other hand strongly resembled the phenotype of the non-mutated Amaretto (Fig. 2), having similar leaf area (8.2% larger), leaf to stem ratio (0.3) and number of tillers (13.7 vs. 15.1) as the non-mutated Amaretto (Table 6, Fig. 2).

**Table 5.** Onset of spike emergence for spring wheat grown in climate chambers in three R:FR light regimes, recorded for 70 days after sowing, *n* = 10.

	Spike emergence		
Mutant, cultivar	R:FR 2.5	R:FR 0.9	R:FR 0.4
	Days ± SE	Days ± SE	Days ± SE
A9	67 ± 2.50	60 ± 4.06	59 ± 4.65
A20	61 ± 1.93	54 ± 0.84	53 ± 1.58
A48	69 ± 1.15	62 ± 2.21	61 ± 0.63
T104	>70	68 ± 0.00	66 ± 2.79
A151	63 ± 2.59	55 ± 1.69	52 ± 2.04
Amaretto	58 ± 4.44	51 ± 2.80	51 ± 2.42
Triso	57 ± 1.91	52 ± 1.85	50 ± 0.97

The mutant lines A9 and T104 had significantly shorter leaf sheaths and laminae than the non-mutated cultivars in all three light treatments (*P* < 0.001, Table 4). These two lines resembled a wheat phenotype with shorter internode segments along its culm that has been characterized as *Rht8* semi-dwarf mutant with reduced cell elongation and reduced sensitivity to brassinosteroids (Gasperini *et al.* 2012). Differences in leaf sheath and lamina length of lines A9 and T104 compared to non-mutated plants were not explicitly triggered by the reduced R:FR ratio. Both lines also had a much larger leaf area than the non-mutated cultivars in near neutral light (A9: 101 % and T104: 70.6 %), but their leaf area was strongly reduced by the FR light (Table 6). Both mutant lines produced thin and pale leaves, with a lower concentration of chlorophyll than the non-mutated cultivars (33.3 % and 52 %, respectively at R:FR 0.9, Table 6, Fig. 2). Line T104 in particular had narrow leaves and a profusely tillering phenotype with many spikes (22 vs. 16 in non-mutated cultivars). Finally, mutant A20 showed no significant difference in elongation behaviour to the non-mutated Amaretto (Table 4). In near-neutral light, the plants were on average only 8.3 % smaller at harvest, had 9.7 % lower total dry biomass and 3.9 % lower chlorophyll concentration in their leaves than the non-mutated Amaretto plants (Table 6, Fig. 2).

**Table 6.** Growth parameters of mutants and non-mutated cultivars grown in three R:FR light regimes in climate chambers. Measures taken 14 weeks after sowing, *n* = 10.

R:FR	Mutant, cultivar	Plant height (cm)	Total dry matter (g)	Stem weight (g)	Leaf weight (g)	Leaf area (cm^2^)	Leaf:stem ratio	Number of spikes	Chlorophyll a:b ratio	Total chlorophyll (µg m^−2^)
	A9	73.0 ± 9.9	42.9 ± 17.1	20.0 ± 6.2	10.5 ± 1.6	1882.6 ± 300.7	0.58 ± 0.20	15.4 ± 5.1	4.7 ± 0.82	0.48 ± 0.24
	A20	85.3 ± 4.5	52.2 ± 10.3	23.8 ± 4.6	8.7 ± 1.4	1273.5 ± 197.2	0.37 ± 0.06	16.5 ± 1.6	3.6 ± 0.31	0.93 ± 0.12
	A48	71.9 ± 5.5	28.1 ± 6.8	15.5 ± 4.5	7.6 ± 2.0	1211.6 ± 88.1	0.53 ± 0.17	12.5 ± 2.7	3.7 ± 0.39	0.74 ± 0.12
2.5	T104	73.4 ± 15.3	32.1 ± 12.6	19.0 ± 7.8	7.9 ± 2.3	1755.6 ± 577.5	0.47 ± 0.18	19.9 ± 10.3	4.2 ± 0.16	0.48 ± 0.08
	A151	80.8 ± 7.1	54.4 ± 13.5	23.9 ± 6.5	8.4 ± 1.9	1053.7 ± 256.5	0.37 ± 0.09	16.2 ± 3.9	3.8 ± 0.26	0.81 ± 0.12
	Amaretto	92.9 ± 6.8	67.4 ± 3.4	24.5 ± 1.7	7.2 ± 1.3	1096.9 ± 101.0	0.29 ± 0.04	16.0 ± 1.8	3.1 ± 0.17	1.05 ± 0.07
	Triso	93.5 ± 6.9	65.9 ± 13.7	23.8 ± 5.2	6.4 ± 1.0	1007.2 ± 167.6	0.28 ± 0.06	16.6 ± 2.3	3.1 ± 0.37	1.07 ± 0.12
	A9	84.7 ± 8.7	58.8 ± 14.1	23.9 ± 4.5	10.3 ± 1.6	1658.1 ± 221.4	0.44 ± 0.07	17.1 ± 1.9	3.7 ± 0.46	0.68 ± 0.19
	A20	86.1 ± 5.6	53.9 ± 12.6	22.1 ± 2.9	7.4 ± 0.7	930.9 ± 107.3	0.34 ± 0.03	16.7 ± 2.2	3.0 ± 0.20	0.98 ± 0.09
	A48	83.2 ± 3.5	36.3 ± 6.9	17.9 ± 3.6	6.7 ± 0.7	910.5 ± 27.5	0.38 ± 0.05	10.5 ± 1.4	3.0 ± 0.21	0.86 ± 0.05
0.9	T104	89.9 ± 5.3	44.1 ± 8.6	23.6. ± 5.1	7.4 ± 1.3	1399.4 ± 229.0	0.31 ± 0.02	22.3 ± 4.8	4.0 ± 0.20	0.48 ± 0.05
	A151	87.5 ± 5.7	52.2 ± 3.6	19.1 ± 2.4	6.3 ± 1.0	892.4 ± 152.2	0.33 ± 0.03	13.7 ± 1.9	3.2 ± 0.22	0.88 ± 0.08
	Amaretto	93.9 ± 7.1	59.7 ± 10.2	18.0 ± 5.3	5.5 ± 1.3	825.1 ± 219.1	0.33 ± 0.14	15.1 ± 3.3	2.9 ± 0.29	1.02 ± 0.12
	Triso	97.3 ± 5.5	64.4 ± 6.6	21.0 ± 2.3	5.5 ± 0.6	820.3 ± 105.0	0.26 ± 0.03	16.3 ± 3.6	3.1 ± 0.15	1.00 ± 0.04
	A9	88.4 ± 6.6	51.9 ± 23.6	22.0 ± 7.2	8.1 ± 1.3	1219.0 ± 94.3	0.39 ± 0.09	13.9 ± 2.3	3.6 ± 0.44	0.57 ± 0.18
	A20	92.7 ± 2.9	62.1 ± 11.9	27.1 ± 5.1	8.3 ± 1.1	1067.5 ± 194.3	0.31 ± 0.05	15.7 ± 2.8	3.0 ± 0.25	0.93 ± 0.14
	A48	86.5 ± 3.4	37.1 ± 6.6	18.9 ± 3.4	6.2 ± 0.9	797.3 ± 46.8	0.33 ± 0.03	9.6 ± 0.5	2.7 ± 0.16	0.87 ± 0.10
0.4	T104	89.7 ± 6.3	39.8 ± 10.3	20.4 ± 4.9	5.4 ± 1.1	880.4 ± 119.1	0.27 ± 0.03	18.0 ± 3.9	3.6 ± 0.27	0.51 ± 0.08
	A151	88.8 ± 4.8	58.7 ± 14.9	20.4 ± 5.6	6.6 ± 1.1	740.5 ± 264.2	0.34 ± 0.09	14.2 ± 3.0	3.0 ± 0.11	0.87 ± 0.08
	Amaretto	100.0 ± 4.1	75.2. ± 6.2	26.1 ± 1.4	6.5 ± 0.6	1008.3 ± 217.1	0.25 ± 0.03	15.2 ± 2.4	2.9 ± 0.24	0.91 ± 0.15
	Triso	99.8 ± 4.5	71.7 ± 9.8	24.2 ± 3.0	5.3 ± 0.8	740.5 ± 120.5	0.22 ± 0.02	14.9 ± 1.9	3.0 ± 0.37	0.92 ± 0.19

In general, added FR light in the climate chambers significantly increased the final height of the plants (10.67 cm, *P* < 0.001) but reduced leaf area (−402.97 cm^2^, *P* < 0.001) and leaf weight (−1.46 g, *P* < 0.001) and thus also the leaf to stem ratio (−0.11, *P* < 0.001, Table 7). Plants in added FR light also showed a significant reduction in the chlorophyll *a:b* ratio (−0.63, *P* < 0.001, Table 7), indicating the enlargement of the chlorophyll antenna size (Webb and Melis 1995; Neidhardt *et al.* 1998).

**Table 7.** Differences in the growth and yield parameters of five selected spring wheat mutant lines and the non-mutated cultivars of their origin in response to different R:FR light regimes and in response to mutation, *n*=10

Measured traits	Compared treatments	Estimate	Std. Error	Pr(>|z|)	Confidence	interval
					lower	upper
Plant height (cm)	R:FR (0.9–2.5)	7.37	1.707	<0.001	4.03	10.72
	R:FR (0.4–2.5)	10.67	1.707	<0.001	7.33	14.02
	cultivar-mutant	12.11	2.268	<0.001	7.67	16.56
Leaf area (cm^2^)	R:FR (0.9–2.5)	-263.20	85.540	0.008	-430.85	-95.55
	R:FR (0.4–2.5)	-402.97	85.380	<0.001	-570.31	-235.62
	cultivar-mutant	-263.18	216.620	0.590	-687.77	161.40
Leaf weight (g)	R:FR (0.9–2.5)	-1.09	0.360	0.009	-1.79	-0.38
	R:FR (0.4–2.5)	-1.46	0.360	<0.001	-2.16	-0.75
	cultivar-mutant	-1.64	0.911	0.232	-3.43	0.14
Stem weight(g)	R:FR (0.9–2.5)	-0.73	1.369	0.959	-3.41	1.96
	R:FR (0.4–2.5)	1.20	1.369	0.802	-1.48	3.89
	cultivar-mutant	1.78	1.848	0.748	-1.84	5.40
Leaf to stem ratio	R:FR (0.9–2.5)	-0.07	0.026	0.022	-0.12	-0.02
	R:FR (0.4–2.5)	-0.11	0.026	<0.001	-0.16	-0.06
	cultivar-mutant	-0.11	0.044	0.035	-0.20	-0.03
Total dry matter(g)	R:FR (0.9–2.5)	3.53	1.731	0.144	0.14	6.93
	R:FR (0.4–2.5)	7.60	1.731	<0.001	4.20	10.99
	cultivar-mutant	22.42	7.520	0.024	5.80	35.28
Total chlorophyll (µg/cm^-2^)	R:FR (0.9–2.5)	0.05	0.034	0.416	-0.02	0.12
	R:FR (0.4–2.5)	0.01	0.034	0.999	-0.06	0.07
	cultivar-mutant	0.26	0.147	0.260	0.03	0.54
chlorophyll a to b ratio	R:FR 0.9	-0.48	0.114	<0.001	-0.71	-0.26
	R:FR 0.4	-0.63	0.114	<0.001	-0.85	-0.41
	cultivar-mutant	-0.50	0.308	0.329	-1.10	0.11
Number of spikes	R:FR 0.9	-0.01	0.042	0.994	-0.10	0.07
	R:FR 0.4	-0.11	0.042	0.048	-0.19	-0.02
	cultivar-mutant	0.03	0.139	0.999	-0.24	0.30

In summary, two of the final mutant lines showed significantly reduced shade avoidance responses in low R:FR light responding to light composition with respect to leaf sheath elongation. Leaf lamina elongation showed reduced responses only in the stronger shade treatment (green filter). Of these two lines, A151 showed a 20.6 % reduced leaf sheath elongation compared to the non-mutated Amaretto plants and is the most promising candidate for further studies. While A48 showed an even stronger reduction in elongation (68.9%), it differed from the non-mutated cultivar in several aspects (extremely low early vigour, delayed development, thicker and shorter leaves and reduced number of tillers). A151, on the other hand, strongly resembled the original cultivar throughout its development, reaching almost the same final height but without a rapid elongation response to shading (Fig. 2).

## Discussion

### Shade avoidance in crops

It could be expected that half a century of breeding for short cultivars with a high reproductive output would already have selected against shade avoidance responses in modern wheat germplasm, since there is a trade-off between elongation responses and yield. However, a study of 20th century Argentinian wheat cultivars showed that selection for higher yield has not reduced shade avoidance responses. On the contrary, the magnitude of the responses is greater in modern than in older cultivars (Ugarte *et al.* 2010). Our results, particularly those from the climate chambers, support these findings.

The original cultivars in our experiments responded with strong elongation to both reduced R and to increased FR light. Thus, it seems that in spite of the introduction of dwarfing genes to more than 70 % of current commercial wheat varieties (Gale *et al.* 1985; Evans 1998) these plants still maintain their ability for rapid elongation in shade. This may not have been of great practical significance because of suppression of shade-induced elongation through the use of stem-shortening growth regulators and routine application of herbicides. This may have lessened the urgency for the development of cultivars with weak shade avoidance responses when grown at high density. Increases in the yields of many cereals achieved over the last 60 years through breeding have been in large part based on selecting short cultivars with higher harvest index and tolerance of high sowing densities (Fischer and Edmeades 2010). Furthermore, under a given set of growth conditions, the yield of a cultivar is not only affected by management and plant density but also by competition for resources from weeds, which are more efficiently suppressed by higher crop densities (Weiner *et al.* 2001). The use of agrochemicals such as stem-shortening growth regulators is being questioned in many countries. Consequently, the development of cultivars with low shade avoidance responses is becoming more desirable and is likely to make a contribution to raising grain yield in future years.

More than 20 semi-dwarfing loci (Rht) and 25 alleles are known to be associated with the semi-dwarf growth habit of wheat (Konzak 1987). Though the reduced height of the plants has allowed the partitioning of more assimilates to the developing grains (Evans 1993), dwarfing alone may not eliminate seed yield loss due to density-triggered, light-induced growth elongation. Thus, the objective of this study was not to reduce the size of the cultivars *per se*, but to diminish the plants’ plasticity in response to light quality, especially at early growth stages, thus reducing the yield reduction caused by shade-induced elongation. In other words, we wish to preserve the height and form of plants grown at low densities when they are grown at the high densities typical of modern cereal farming.

In recent decades, much progress has been made in understanding the complex interactions of light receptors, transcription factors and plant hormones involved in shade avoidance through studying mutants of *Arabidopsis thaliana*. In addition to phytochromes, blue light sensing cryptochromes and phototropins and plant hormones, notably gibberellic acid, ethylene, brassinosteroids and auxin also play a role in regulating the growth and development of shaded plants (McCormac *et al.* 1992; Chen *et al.* 2004; Pierik *et al.* 2004; Vandenbussche *et al.* 2005; Franklin 2008; Xu *et al.* 2015; de Wit *et al.* 2016). However, extending the findings of a dicot model species such as *Arabidopsis* to monocot crop plants presents major challenges. Extensive genome rearrangements, duplication events and lost genes between monocot and dicot lineages render the identification of orthologous relationships difficult (Bennetzen 2007). While many eco-physiological experiments on the effects of shade avoidance have been conducted in crop species (Ballaré *et al.* 1987, 1991; Ghersa *et al.* 1994), relatively little is known about the genetic light signal transduction networks in monocots (Mathews and Sharrock 1996; Sawers *et al.* 2005), particularly in allohexaploid wheat.

### Light treatments for shade avoidance experiments

The spectral light environment changes gradually during crop growth. Sunlight contains blue, red and far-red light at high intensities and the R:FR ratio is just above one (Gundel *et al.* 2014). When plants start to sense near neighbours, they are responding to a R:FR ratio that has decreased to <1 due to reflection of FR from nearby plants (de Wit *et al.* 2016). In dense shade, the R:FR can be as low as 0.2 or less (Gundel *et al.* 2014). As competition for light increases, the canopy closes and PAR decreases, blue light will decrease to levels in which signalling through the blue light-absorbing cryptochrome occurs (de Wit *et al.* 2016). Thus, during crop establishment, the entire plant is exposed to R:FR >1 but when the canopy closes, the ratio gradually decreases due to first reflection from neighbours, and later, to transmission of light through leaves. However, cereal crops are grown in open environments so the top of the canopy will always be exposed to a high R:FR ratio. This complicates matters when it comes to simulating shade in experimental setups.

In our first two screenings we used green filters resulting in modest decrease in the R:FR ratio (to 0.65). This eliminated the most severely dwarfed mutants and reduced the number to a manageable set of 36 for more detailed selection experiments. To further separate mutants with different shade avoidance responses, morphological studies were performed, and the green filter was changed to decrease the R:FR ratio to 0.16. A neutral grey filter with the same reduction of PAR was used as control.

In both the screening and selection experiments, the green filter decreased blue irradiance in the top of the canopy and this could also have enhanced the response to low R:FR (de Wit *et al.* 2016). However, with a very large number of mutants to deal with at the initial stages this compromise was considered acceptable. In the last experiment where only five mutants and two parent cultivars were involved, it became possible to avoid any blue light effect by enhancing FR intensities directly in climate chambers using metal halide lamps with a higher R:FR ratio than natural sunlight. This ensured a maximum photo-conversion of the phytochrome system from the physiologically inactive P_r_ form that absorbs red light to the physiologically active P_fr_ form that absorbs FR and prevents FR induced elongation growth (Xu *et al.* 2015). The use of LED modules to create a low R:FR ratio enabled us to create light environments with similar PAR and maintain high irradiance of blue wavelengths on the top of the canopy, while creating three levels of R:FR.

In the field, an entire plant will experience reduction of R:FR and blue light from the top of the canopy only if it is growing under a tall canopy. When working with crops that are grown in full sun, it would be difficult if not impossible to artificially create a light environment where both the R:FR ratio and blue light are high at the top of the canopy. Therefore, as in all controlled experiments using filters or FR light sources, our results may be slightly stronger than those we would expect in the field. We know only one previous study of how a gradient of R:FR down through a canopy affects shoot elongation (Weijschedé *et al.* 2006)

### Reducing shade avoidance responses

Two successful strategies used to investigate shade avoidance have been the overexpression of phytochrome photoreceptors (Keller *et al.* 1989; Clough *et al.* 1995; Jordan *et al.* 1995) and the study of phytochrome deficient plants (Whitelam and Smith 1991; Childs *et al.* 1997; Sawers *et al.* 2002; Sheehan *et al.* 2007). Due to the intricate light and hormonal signalling networks, these approaches carry a high probability of undesirable pleiotropic effects. Furthermore, morphological responses to heterologous expression of *PHY* phytochrome genes can vary strongly between (Sawers *et al.* 2005) and within species (Kay *et al.* 1989; Nagatani *et al.* 1991; Kebrom and Brutnell 2007), making it difficult to apply conclusions drawn from one species to another.

There have been to date few studies on phytochromes and the transgenic overexpression of phytochrome photoreceptors in wheat (Carr-Smith *et al.* 1994). It has been shown that the overexpression of the oat *PHYA* gene in wheat inhibits coleoptile elongation in continuous FR light, increases the synthesis of anthocyanin and promotes leaf unrolling (Shlumukov *et al.* 2001; Sineshchekov *et al.* 2001). There is no information available concerning how these transgenic plants develop and what growth characteristics they possess, however.

Reduced leaf sheath and lamina lengths in low R:FR light have also been used to measure effects in tobacco and tomato overexpressing oat *PHYA* (Boylan and Quail 1989; Robson *et al.* 1996) and in rice mutants overexpressing the Arabidopsis *PHYA* (Garg *et al.* 2006). A drawback in many of these transgenic shade avoidance phenotypes has been the severe dwarfism caused by such major modifications of primary light sensors. For these reasons, we discarded strong dwarfing phenotypes in the early steps of the screening process and only selected mutants with normal height. This gave us four out of five selected mutant lines that showed reduced elongation of the first leaf sheath or second leaf lamina in added FR light, compared with non-mutated plants. Several studies have shown that elongation responses to low R:FR ratios are predominantly mediated by phytochrome A and B, as mutants deficient in phyA or phyB display dramatically elongated phenotypes with early flowering (Nagatani *et al.* 1991; Devlin et al. 1992, 1997; Halliday *et al.* 1994; Yanovsky *et al.* 1995). However, little is yet known about effectors of light signalling downstream to the primary light receptors in monocots, especially in wheat.

Future molecular studies of the mutants obtained from our experiments may reveal novel molecular handles, which can be used to modify responses to changed light distributions. This approach presents fewer pleiotropic complications than does modification of primary light receptors. Testing the performance of these lines in field experiments, especially in high-density cultivation systems, will show if the reduced elongation in response to neighbours will result in the desired increases in grain yield.

## Conclusions

Shade avoidance can be disadvantageous for crop plants, because it reduces allocation of resources to reproductive yield, increases the risk of lodging, and reduces weed suppression at high crop density. We succeeded in producing lines of spring wheat with reduced shade avoidance using a forward approach with induced mutations and phenotypic screening. These mutants may be useful in developing new wheat varieties with reduced shade avoidance responses, and in generating molecular handles to modify the reaction of plants to changed light quality.

## Sources of Funding

This research was supported by the Program of Excellence of the University of Copenhagen.

## Contributions by the Authors

S.B.A. and W.W. designed the study. W.W., S.B.A. and E.R. performed the experiments. C.B.P. and W.W. did the statistical analyses. J.W. obtained the funding. All authors contributed to writing the article.

## Conflicts of Interest

None declared.
